# ﻿Reticulation within *Sporobolus*: recognition of two new sections, *Acinifolii* and *Thellungia*, and a new genus, *Hyalolemma* (Poaceae, Chloridoideae, Zoysieae, Sporobolinae)

**DOI:** 10.3897/phytokeys.261.157741

**Published:** 2025-08-11

**Authors:** Paul M. Peterson, Konstantin Romaschenko, Robert J. Soreng, Yolanda Herrera Arrieta

**Affiliations:** 1 Department of Botany MRC-166, National Museum of Natural History, Smithsonian Institution, Washington, DC 20013-7012, USA National Museum of Natural History, Smithsonian Institution Washington United States of America; 2 Instituto Politécnico Nacional, CIIDIR Unidad-Durango-COFAA, Durango, C.P. 34220, Mexico Instituto Politécnico Nacional, CIIDIR Unidad‐Durango‐COFAA Durango Mexico

**Keywords:** Classification, ITS, lectotypification, phylogeny, plastid DNA sequences, Poaceae, *Sporobolus*, systematics, taxonomy

## Abstract

We present a molecular DNA phylogeny utilizing four plastid regions (*rps16–trnK* spacer, *rps16* intron, *rpl32–trnL* spacer, *ndhA* intron) and the nuclear ribosomal internal transcribed spacer (ITS) region, investigating 123 species of subtribe Sporobolinae. We also aimed to assess the generic limits of *Sporobolus*, characterize possible subgeneric relationships among species in the genus, and identify hypothesized reticulation events. The core Bayesian tree, based on combined and congruent plastid and ITS regions, is well resolved, and 11 sections within a monophyletic *Sporobolus* are strongly supported. We describe a new genus, *Hyalolemma*, with two species and include a key; erect two new sections within *Sporobolus*, S.sect.Acinifolii and S.sect.Thellungia; and make three new combinations, *Hyalolemmacompactum*, *H.somalensis*, and *Sporoboluscollinus*. The names *Eragrostiscollina* Trin. and *Sporoboluscompactus* Clayton are lectotypified.

## Abstract (es)

Presentamos una filogenia molecular de ADN utilizando cuatro regiones plastidiales (espaciador *rps16-trnK*, intrón *rps16*, espaciador *rpl32-trnL* e intrón *ndhA*), así como la región nuclear del espaciador transcrito interno ribosomal (ITS) para investigar 123 especies de la subtribu Sporobolinae. También nos propusimos evaluar los límites genéricos de *Sporobolus*, caracterizando posibles relaciones subgenéricas entre las especies del género e identificando eventos hipotéticos de evolución reticulada. El árbol bayesiano principal, derivado de la combinación de regiones plastidiales e ITS congruentes, presenta una buena resolución. Se respaldan firmemente las 11 secciones dentro de un *Sporobolus* monofilético. Describimos un género nuevo, *Hyalolemma*, con dos especies incluyendo una clave; establecimos dos nuevas secciones dentro de *Sporobolus*, S.sect.Acinifolii y S.sect.Thellungia; y creamos tres nuevas combinaciones, *Hyalolemmacompactum*, *H.somalensis*, y *Sporoboluscollinus*. Los nombres *Eragrostiscollina* Trin. y *Sporoboluscompactus* Clayton fueron lectotipificados.

## ﻿Introduction

The genus *Sporobolus* R. Br. (dropseed) includes approximately 220 species worldwide and is placed in subtribe Sporobolinae Benth., tribe Zoysieae Benth., and subfamily Chloridoideae Kunth ex Beilschm. (Soreng et al. 2022). *Sporobolus* is characterized by single-flowered spikelets (rarely 3–27-flowered), 1-veined (occasionally 3-veined) lemmas, fruits with free pericarps (commonly swelling and mucilaginous when wet, forcibly ejecting the seed), and ligules that are a line of hairs or a ciliate membrane (Peterson et al. 2003, 2004, 2014a; Giraldo-Cañas and Peterson 2009).

The most compelling subgeneric classification of *Sporobolus* was based on a phylogeny derived from DNA sequence data (of 144 *Sporobolus* species), using four plastid regions (*rpl32–trnL*, *ndhA*, *rps16–trnK*, *rps16*) and one nuclear marker (ITS), ultimately recognizing 11 sections and 11 subsections (Peterson et al. 2014a, b). This classification was somewhat controversial, since it subsumed within *Sporobolus* three long-established genera: *Calamovilfa* (A. Gray) Hack. ex Scribn. & Southw., *Crypsis* Aiton, and *Spartina* Schreb.; and two multi-flowered species: *Eragrostisadvena* (Stapf) S.M. Phillips [≡ *Thellungiaadvena* Stapf ≡ *Sporobolusadvenus* (Stapf) P.M. Peterson] and *E.megalosperma* F. Muell. ex Benth. [≡ *S.megalospermus* (F. Muell. ex Benth.) P.M. Peterson]. A recent phylogenomic analysis of *Sporobolus*, using nuclear Angiosperm353 probes and whole plastomes based on a smaller sample (16 species), confirmed the monophyly of *Sporobolus*, with *Spartina* [S.sect.Spartina (Schreb.) P.M. Peterson & Saarela] and *Crypsis* [C.sect.Crypsis (Aiton) P.M. Peterson] as derived clades within *Sporobolus* (GPWG III 2024). It is interesting to note that Stapf (1920) indicated that the new species (*Thellungiaadvena*) found among wool refuse near the Derendingen Mill in Switzerland “was found to be very like that of a *Sporobolus* but distinguished by the presence of several (mostly three and sometimes four) florets in each spikelet.”

We continue to advance the concept of core phylogeny and its usefulness, especially in phylogenetic studies of large genera with complex relationships among their members using conventional genetic data such as cpDNA and ITS nrDNA sequences. We consider a core phylogeny to represent an evolutionary pattern among species based only on direct descent, excluding taxa or individuals with genomes of multiple origins. Following this concept in our phylogenetic studies, we split the analysis into two main phases. First, we develop a phylogenetic tree using only the “core” set of available taxa or individuals presumably having a single origin. Then, we rerun the analysis with the addition (usually one taxon at a time) of taxa or individuals with genetic data (plastid or nuclear) for which multiple origins were detected. This taxon duplication approach (Pirie et al. 2008; Pelser et al. 2010; Soreng et al. 2010; Peterson et al. 2015a, 2016, 2020, 2021, 2025) uses the core phylogeny as a framework to test the affinities of species based on different types of genetic data. Eventually, these affinities can be characterized, providing inferences about species origins and geographical distribution. In most cases, a core phylogeny demonstrates a better-developed topology of the species and stronger support for phylogenetic groups compared to previous studies, as seen in *Agrostis* L. and *Calamagrostis* Adans. (Saarela et al. 2017; Peterson et al. 2021, 2025). We attribute this to the elimination of incongruent data from analysis, including confounding ITS data that likely result from incomplete genomic introgression, gene flow, or incomplete concerted evolution. The tendency of nrDNA to homogenize during different stages of genomic introgression and to reflect to varying degrees the affinities with parental species is well documented (Fuertes Aguilar et al. 1999; Bailey et al. 2003; Liu et al. 2020; Wang et al. 2023). Compared to low-copy gene analysis, ITS data can sometimes indicate an intermediate position for hybrid species, i.e., between the locations of presumable parental taxa (Romaschenko et al. 2013), or provide an erroneous phylogenetic position for such species due to putative long branch attraction (Peterson et al. 2021). Of particular interest is the formation of strongly supported ITS groups with shared morphological features that encompass species with divergent plastid lineages (Peterson et al. 2021). We identify these as “floating ITS groups” because they often show little affinity to other clades, while their inclusion in the analysis may weaken backbone support (e.g., the *Deschampsiagrostis* group of *Calamagrostis*; see Peterson et al. 2021). Though the origin of floating ITS groups is ambiguous, it might involve the following processes: hybridization between species representing distant lineages; extensive gene flow between hybrid and parental populations; incomplete genomic introgression and formation of confounding (via homogenization) ITS sequences; separation and geographical isolation of individuals with similar confounding ITS sequences and distinct plastid sequences; reduction of gene flow; and the formation of new species and subsequent evolutionary diversification. We found it useful to test our phylogenies for the presence of floating ITS groups, since these clades often share morphological characteristics that are important descriptors of their evolutionary history (Wang et al. 2023).

In our previous phylogeny of the Sporobolinae (Peterson et al. 2014a), we listed two *incertae sedis* categories: one species, *Sporobolussomalensis* Chiov., outside of *Sporobolus*, and 13 species with uncertain affinities within the genus. In this paper, we take a closer look at the basal lineage consisting of *Sporobolusacinifolius* Stapf, *S.albicans* Nees, and *S.tenellus* (A. Spreng.) Kunth. We also track species that exhibit incongruent ITS versus plastid marker alignment, such as *S.consimilis* Fresen., *S.robustus* Kunth, *S.scabridus* S.T. Blake, and *S.tourneuxii* Coss.; and species in S.sect.Crypsis and S.subsect.Subulati P.M. Peterson. Additionally, we include two more multi-flowered species, *Eragrostiscollina* Trin. from Persia and *Sporobolusramigerus* (F. Muell.) P.M. Peterson, Romasch. & R.L. Barrett from Australia, to test their affinities, and include *Sporoboluscompactus* Clayton, an ally of *S.somalensis* (Clayton 1970; Barrett et al. 2020).

## ﻿Material and methods

### ﻿Taxon sampling

We sampled 135 individuals representing 122 species (55%) of *Sporobolus*. A complete list of taxa, including authorities, voucher information, and GenBank numbers, is presented in Appendix 1. Most of these DNA sequences were previously published in GenBank and were initially used in Peterson et al. (2014a). We include 18 new sequences in GenBank, representing two species, *Eragrostiscollina* and *Sporobolusniliacus* (Fig. & De Not.) P.M. Peterson, both extracted from herbarium specimens housed in the United States National Herbarium (US).

We designed our study to characterize relationships among species of *Sporobolus* and relatives, principally in the Sporobolinae Benth. (including *Psilolemma* S.M. Phillips), and including an outgroup from the Zoysiinae Benth. (*Urochondra* C.E. Hubb. and *Zoysia* Willd.) (Peterson et al. 2014a; Soreng et al. 2022).

### ﻿Phylogenetic methods

All procedures related to the sequencing of the plastid and ITS regions were performed in the Laboratory of Analytical Biology at the Smithsonian Institution. Detailed methods for DNA extraction, amplification, and sequencing are given in Romaschenko et al. (2012) and Peterson et al. (2010a, b, 2012, 2014a, 2015a, b, 2016). We used Geneious Prime v.2020.1.4 (Kearse et al. 2012) for contig assembly of bidirectional sequences of the *rps16–trnK* spacer, *rps16* intron, *rpl32–trnL* spacer, *ndhA* intron, and ITS regions, and MUSCLE (Edgar 2004) to align consensus sequences and adjust the final alignment. Phylogenetic trees were constructed from the three combined cpDNA and nrDNA datasets (see Appendix 1) using partitioned maximum likelihood analysis implemented in IQ-TREE 2 (Minh et al. 2020; Chernomor et al. 2016). The best-fit evolutionary models for partitions were inferred using ModelFinder Plus (MFP; Kalyaanamoorthy et al. 2017) based on the Akaike Information Criterion. In the partition model used for maximum likelihood analysis, we specified a substitution model for each DNA region (*rps16–trnK* spacer, *rps16* intron, *rpl32–trnL* spacer, *ndhA* intron, and ITS; see Table 1), allowing each partition to have its own evolutionary rate. Support was assessed using the approximate Bayes test (aBayes; Anisimova et al. 2011) and 10,000 bootstrap replicates (BS; Felsenstein 1985). Support values with aBayes ≥ 0.95 and BS ≥ 95% were interpreted as strong support.

**Table 1.** Characteristics of the five regions, *rps16-trnK, rps16* intron, *rpL32-trnL*, *ndhA* intron, ITS, and parameters used in phylogenetic analyses indicated by Akaike information criterion (AIC).

	rps16-trnK	rps16 intron	rpL32-trnL	ndhA intron	Combined plastid data	ITS	Overall
Total aligned characters	975	964	937	1236	4112	798	4910
Number of sequences/success	118 (87.4%)	119 (88.1%)	135 (100%)	99 (73.3%)	471 (87.2%)	135 (100%)	606 (89.8%)
Parsimony informative sites	166	134	194	212	706	401	1107
Optimal log-likelihood	-3803.1	-3396.8	-4442.9	-5304.4		-12989.6	
Substitution model	TVM+F+G4	GTR+F+ I+I+R2	TPM3u+F+ I+I+R2	TPM3u+F+ I+I+R2		SYM+I+G4	

### ﻿Principles and phases of phylogenetic reconstruction

**Figure 1.**
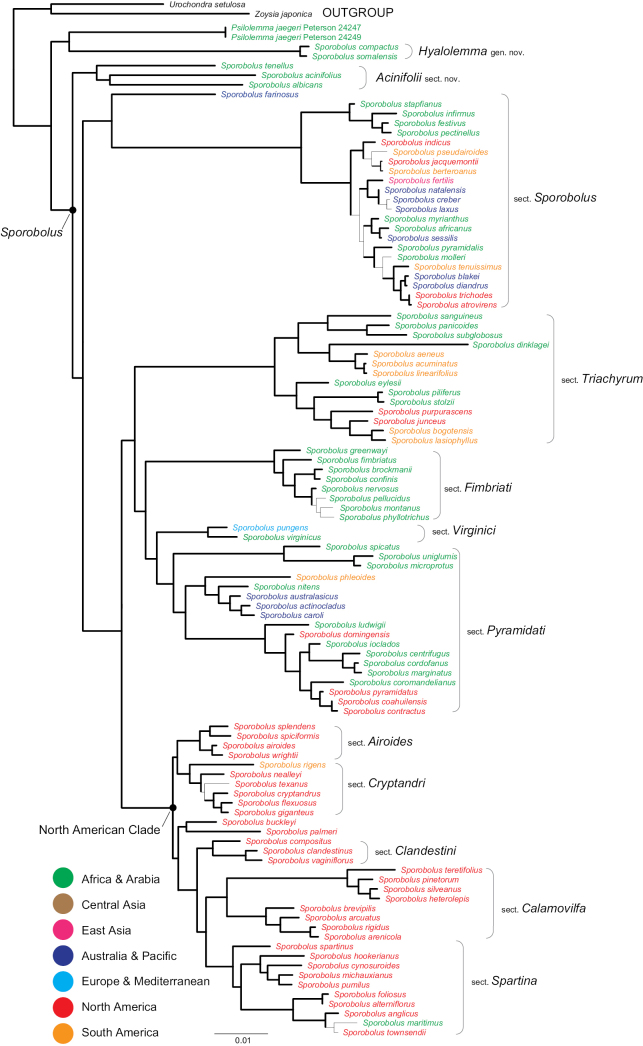
Core Bayesian tree inferred from combined plastid (*rpl32-trnL, ndhA, rps16-trnK, rps16*) and ITS sequences of the Sporobolinae with *Hyalolemma* and *Psilolemma* and *Sporobolus* showing sectional classification including S.sect.Acinifolii with geographic distribution (color). Thick black branches in the phylogram indicate a bootstrap of 95–100 and/or an aBayes of 0.95–1.00. Scale bar = 1% substitutions per site.

**Figure 2.**
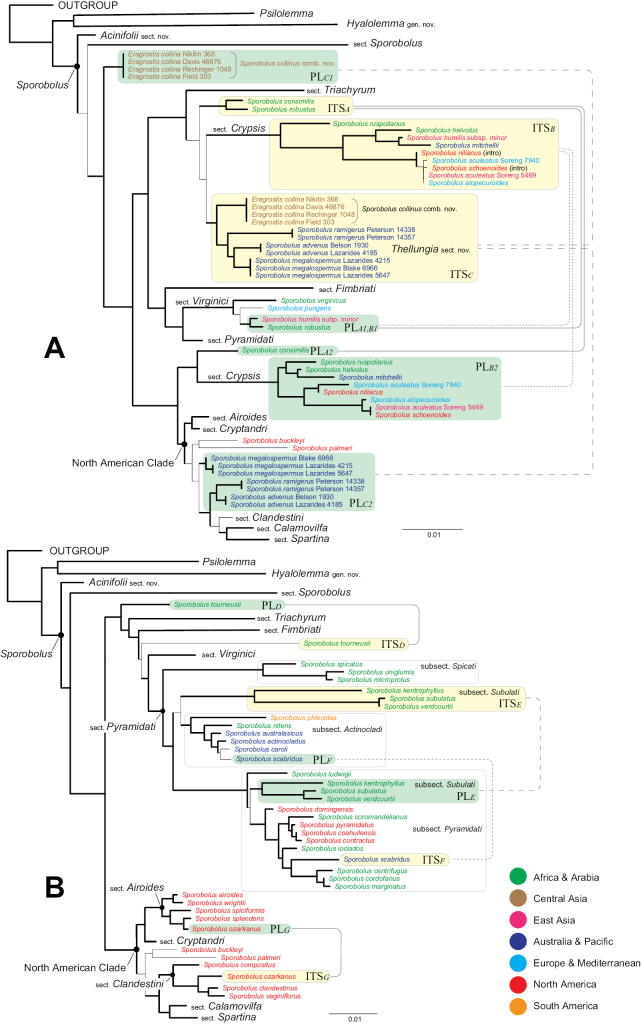
Reticulate origins of species within *Sporobolus* showing sectional classification and geographic distribution (color). Thick black branches in the phylogram indicate a bootstrap of 95–100 and/or an aBayes of 0.95–1.00. Scale bar = 1% substitutions per site. A. Origins of *Eragrostiscollina* (= *Sporoboluscollinus*), *S.advenus*, *S.megalospermus*, and *S.ramigerus* (= S.sect.Thellungia); Sporobolussect.Crypsis, *S.consimilis, S.humilis* subsp. *minor, S.oxylepsis*, and *S.robustus*; B. Origins of *Sporoboluskentrophyllus, S.subulatus, and S.verdcourtii* (= S.subsect.Subulati); *S.ozarkanus, S.scabridus*, and *S.tourneuxii*.

Phylogenetic reconstructions were performed in three main phases. The first phase included preliminary Bayesian and bootstrap analyses (trees from preliminary searches not shown), designed to detect hard incongruences between ITS and plastid data; construction of the preliminary core phylogeny; individual testing of specimens with incongruences against the preliminary core phylogeny; and construction of the final core phylogeny (Fig. 1). The second phase (Fig. 2) included a series of individual searches for the incongruent species to identify the correct affiliation of their ITS and plastid sequences, added independently to the core matrix using the taxon duplication approach. The third phase involved identifying patterns among discordant splits between the ITS and plastid data and constructing overall phylogenies representing putative affinities of the incongruent ITS and plastid sequences tested against the core phylogeny. Procedurally, the three-phased core phylogenetic analysis using the taxon duplication approach is described in Peterson et al. (2025).

### ﻿Assessment of incongruence and data-combining strategy

Combining all congruent data provides better resolution of phylogenetic trees, strengthens support for nodes, and maximizes the informativeness and explanatory power of the character data used in the analysis (Huelsenbeck and Cunningham 1996). The plastid and ITS topologies resulting from Bayesian and bootstrap analyses were inspected for conflicting nodes with support values of aBayes ≥ 0.95 and/or BS ≥ 95%. If no supported incongruences were found, plastid and ITS sequences were combined and used in the core phylogenetic analysis (Fig. 1). This analysis (combined plastid and ITS sequences) included a subset of *Sporobolus* species representing 11 sections and 11 subsections classified in Peterson et al. (2014a), two unclassified species of *Sporobolus* (*S.compactus* and *S.somalensis*), *Psilolemmajaegeri* (sister to *Sporobolus*), *Eragrostiscollina*, and outgroups *Zoysiajaponica* Steud. and *Urochondrasetulosa* (Trin.) C.E. Hubb. (Zoysiinae). These two outgroup species were selected because they occur within the Zoysieae and have been shown to be sister to the Sporobolinae (Peterson et al. 2010a, 2014a).

### ﻿Taxon duplication approach

A taxon duplication approach (Pirie et al. 2008; Gillespie et al. 2010; Pelser et al. 2010; Soreng et al. 2010; Peterson et al. 2015a, 2016, 2020, 2021, 2025) was applied to 26 specimens representing 19 species for which incongruence between plastid and ITS data was detected (sets 2A and 2B, Appendix 1). Each of these specimens was assigned two entries in the matrices: one containing only ITS and one containing only plastid sequences. This technique allowed us to identify the placements of the incongruent ITS and plastid sequences in the context of the *Sporobolus* core phylogeny. To avoid mutual influence of confounding ITS sequences, each specimen was analyzed separately before being assigned to one of two expanded datasets (sets 2A and 2B), each including samples with a similar pattern of incongruence between ITS and plastid data representing characteristic ITS/plastid discordant splits. The number of specimens added to the core dataset (dataset 1: 105 specimens) to form the extended datasets was as follows: dataset 2A – 20 specimens; dataset 2B – 10 specimens. The outgroups in the expanded sets included the same species used in the core phylogeny (Fig. 1).

We used this taxon duplication approach to resolve our phylogenetic tree, minimizing the diffusing effects of taxa with strongly supported incongruence between plastid and ITS data, while still showing the placements of the plastid and ITS sequences in relation to the taxa in the core phylogeny. This allowed us to hypothesize multiple origins and elucidate complex evolutionary histories within phylogenetic groups.

## ﻿Results

### ﻿Phylogenetic analyses

Eighteen sequences (18/608 = 3%) in our study are newly reported in GenBank, and 97% (590/608) are previously published sequences (Appendix 1) generated for earlier studies (Peterson et al. 2010a, 2012, 2014a). Ten point four percent (70/671) of the sequences (ITS and plastid) in our dataset are missing. Total aligned characters for individual regions and other parameters are shown in Table 1.

### ﻿Core phylogeny

The core Bayesian tree, based on combined, congruent plastid regions (*rps16–trnK* spacer, *rps16* intron, *rpl32–trnL* spacer, *ndhA* intron) and ITS, is well resolved, and 11 sections within a monophyletic *Sporobolus* (including the new section Acinifolii) are strongly supported (BS = 95–100; aBayes = 0.95–1.00) (Fig. 1). The first split within *Sporobolus* includes three species–*Sporobolusacinifolius*, *S.albicans*, and *S.tenellus* (S.sect.Acinifolii, the new section) from Africa–which are sister to the remaining species in the genus. The next split includes species in sect. Sporobolus [including the type *S.indicus* (L.) R. Br.] from Africa, Australia, and the Western Hemisphere, and these are sister to the remaining species. The following split consists of two strongly supported clades (BS = 95–100; aBayes = 0.95–1.00): one with four sections, resolving as (*Triachyrum* (Hochst. ex A. Braun) Veldkamp (*Fimbriati* Veldkamp (*Pyramidati* P.M. Peterson + *Virginici* Veldkamp))), sister to a clade of five sections, resolving as (*Airoides* (Torr.) P.M. Peterson + *Cryptandri* P.M. Peterson) (*Clandestini* P.M. Peterson (*Calamovilfa* (A. Gray) P.M. Peterson + *Spartina* (Schreb.) P.M. Peterson & Saarela)). The latter five-section clade includes species mainly from North America (the “North American clade”), whereas the four-section clade includes species from Africa, the Western Hemisphere, Australia, and Europe.

Sister to *Sporobolus* in our core tree is a strongly supported clade (BS = 97, aBayes = 0.95) that includes two accessions of *Psilolemmajaegeri* + (*Sporoboluscompactus* + *S.somalensis*) (Fig. 1). The latter two species are genetically variable, with two nucleotide substitutions in ITS, seven nucleotide substitutions in the plastid markers, and five insertion/deletion events in the plastid markers, ranging from 2–46 nucleotide gaps. They are morphologically distinct, differing in lower glume shape and length and in upper glume length.

### ﻿Phylogenetic trees with taxon duplication (including species with incongruent ITS and plastid sequences)

The taxon duplication trees (Fig. 2A, B) provide insight into plastid- and ITS-based relationships among *Sporobolus* species with incongruent plastid and ITS data.

Based on plastid markers, four accessions of *Eragrostiscollina* (PLC1) form a clade sister to the remaining Sporobolus species after the split of sect. Sporobolus (Fig. 2A). Based on ITS, these same four accessions of *E.collina* are sister to *S.ramigerus* (*S.advenus* + *S.megalospermus*). Together, these four species form a strongly supported clade (ITSC), which is weakly supported as sister to Sporobolussect.Crypsis (Aiton) P.M. Peterson (ITSB). Two members of sect. Crypsis collected in North America, *Sporobolusniliacus* (Fig. & De Not.) P.M. Peterson (Baja California, Mexico) and *S.schoenoides* (L.) P.M. Peterson (California, USA), are introductions. The native distribution of species in sect. Crypsis is Africa, Arabia, and Asia. The *E.collina* + (*S.ramigerus* (*S.advenus* + *S.megalospermus*)) clade is morphologically distinct in having multi-flowered spikelets, unlike most *Sporobolus* species. Based on plastid markers, *S.megalospermus* and *S.advenus* + *S.ramigerus* (PLC2) form a grade between the *S.buckleyi* Vasey + *S.palmeri* Scribn. clade and sect. Clandestini (Fig. 2A), all native to North America (American clade). Based on ITS, the *S.consimilis* + *S.robustus* clade (ITS*_A_*) is placed between sects. *Triachyrum* and *Crypsis* (Fig. 2A). Based on plastid data, *S.robustus* is sister to S.humilissubsp.minor Veldkamp (PL*_A1_*_, *B1*_) and is embedded in sect. Virginici, with S.humilissubsp.minor coming from sect. Crypsis (ITS*_B_*), whereas *S.consimilis* aligns as sister to sect. Crypsis (PL*_B2_*).

Based on plastid markers, *S.tourneuxii* (PL*_D_*) is sister to four sections resolving as (*Triachyrum* (*Fimbriati* (*Pyramidati* + *Virginici*))), whereas based on ITS (ITS*_D_*), *S.tourneuxii* is sister to sects. *Virginici* + *Pyramidati* (Fig. 2B). The *S.kentrophyllus* (K. Schum. ex Engl.) Clayton + (*S.subulatus* Hack. −*S.verdcourtii* Napper) clade aligns in different locations within S.sect.Pyramidati: sister to subsect. Actinocladi P.M. Peterson (ITS*_E_*) and embedded within subsect. Pyramidati P.M. Peterson (PL*_E_*). Likewise, *S.scabridus* S.T. Blake aligns in different locations within S.sect.Pyramidati: sister to *S.caroli* Mez in subsect. Actinocladi (PL_*F*_) and sister to *S.centrifugus* (Trin.) Nees+ *S.cordofanus* (Hochst. ex Steud.) Coss. −*S.marginatus* Hochst. ex A. Rich. (ITS_*F*_) in subsect. Pyramidati. *Sporobolusozarkanus* Fernald [≡ S.vaginiflorusvar.ozarkanus Fernald] aligns in different locations: sister to *S.splendens* Swallen (PL*_G_*) in sect. Airoides and sister to *S.clandestinus* (Biehler) Hitchc. + *S.vaginiflorus* (Torr. ex A. Gray) Alph. Wood (ITS*_G_*) in sect. Clandestini.

## ﻿Taxonomy

### ﻿ Hyalolemma

Taxon classificationPlantaePoalesPoaceae

P.M.Peterson, Romasch. & Soreng, gen. nov.

7039D922-B982-5776-9BD1-1E9226AFA119

urn:lsid:ipni.org:names:77367026-1

#### Type.

*Hyalolemmasomalensis* (Chiov.) P.M. Peterson, Romasch. & Soreng ≡ *Sporobolussomalensis* Chiov.

#### Description.

Cushion forming ***perennials***, arising from stout, branching stolons densely clothed in imbricate cataphylls below, innovations extravaginal. ***Culms*** 6–30 cm tall, erect. ***Leaf sheaths*** open for most of their length, glabrous or with pustulate-based hairs, the hairs up to 3 mm long, hyaline; ***ligules*** ≤ 0.–4 mm long, a line of hairs; ***blades*** 0.5–8 cm long, 1.2–2.2 mm wide, flat, stiff, glaucus, and pungent, sometimes with pustulate hairs scattered along the margin. ***Inflorescence*** a panicle 2–13 cm long, 2–7 cm wide, ovate, diffuse, branches capillary. ***Spikelets*** 1.2–2.2 mm long, 1-flowered, lanceolate, laterally compressed to subterete; ***glumes*** shorter to as long as spikelet, hyaline; ***lower glumes*** 0.3–1.2 mm long, orbicular to narrowly oblong, apex obtuse to erose; ***upper glumes*** 0.7–2 mm long, 1-veined, oblong, apex obtuse; ***lemmas*** 1.2–2 mm long, oblong to ovate, 1- or 3-veined, when 3-veined the lateral veins only visible on lower ¼ to ½, hyaline, apex obtuse to truncate, often erose and minutely ciliate; ***paleas*** 2-veined. ***Flowers*** perfect; ***lodicules*** 2; ***anthers*** 1–2 mm long, 3, purplish; ***ovary*** glabrous. ***Caryopses*** 1.2–1.6 mm long, elliptic, brownish with a free pericarp.

#### Etymology.

The name is derived from the Greek “hyalos,” meaning hyaline or transparent, combined with lemma (Greek).

#### Distribution.

*Hyalolemma* comprises two species found in northeastern Africa in Ethiopia and Somalia.

### ﻿Key to the species of *Hyalolemma*

**Table:** 

1	Lower glumes 0.3–0.5 mm long, orbicular; upper glumes as long as the spikelet	***H.compactum***
–	Lower glumes 0.5–1.2 mm long, narrowly oblong; upper glumes 1/2–2/3 as long as the spikelet	***H.somalensis***

### ﻿ Hyalolemma compactum

Taxon classificationPlantaePoalesPoaceae

(Clayton) P.M.Peterson, Romasch. & Soreng, comb. nov.

586DDD9D-7C0B-502F-A90F-1BFFBC679691

urn:lsid:ipni.org:names:77367028-1

Sporobolus compactus Clayton, Kew Bull. 25(2): 248. 1971. Type: Somalia [British Somaliland], Erigavo, 5500 ft, ubiquitous on plains, 26 Sep 1938, *A.S. Mckinnon S.89* (lectotype, designated here: K000365246 [image!]; isolectotypes: K000365245 [image!], US-1815148!).

### ﻿ Hyalolemma somalensis

Taxon classificationPlantaePoalesPoaceae

(Chiov.) P.M.Peterson, Romasch. & Soreng, comb. nov.

55A52384-D079-5477-8593-6D62667C89D1

urn:lsid:ipni.org:names:77367029-1

Sporobolus somalensis Chiov., Annuario Reale Ist. Bot. Roma 6: 169. 1896. Type: Somali Ogaden, 7 Aug 1891, *L. Robecchi-Brichetti 485* (holotype: FT000424 [image!]; isotype: G00022752 [image!]).
= Sporobolusvariegatus Stapf, Kew Bull. 1907: 218. 1907. Type: Somalia, Somaliland, found occasionally in small quantities between Veoholle and Upper Sheikh, Jul 1903, [Lieutenant Colonel] *Appleton s.n.* (holotype: K000365243 [image!]).

### ﻿ Sporobolus sect. Acinifolii P.M.Peterson, Romasch. & Soreng, sect. nov.

Taxon classificationPlantaePoalesPoaceae

18C55241-AAA3-5843-B483-B49D899FFF17

urn:lsid:ipni.org:names:77367030-1

#### Type.

*Sporobolusacinifolius* Stapf, Fl. Cap. 7: 581. 1900.

#### Description.

Mat forming ***perennials*** with elongated and much branched or short rhizomes, innovations intravaginal. ***Culms*** 6–43 cm long with 1–5 nodes, glabrous. ***Leaf sheaths*** open for most of their length; ***ligules*** 0.2–0.3 mm long, a line of hairs; ***blades*** 0.5–12 cm long, 1–3 mm wide, flat, glabrous, apex obtuse to acuminate, occasionally with cartilaginous margins. ***Inflorescence*** a panicle 2–15 cm long, open with effuse to capillary or sometimes dichotomously branched or spiciform with loosely ascending branches. ***Spikelets*** 1–2.5 mm long, 1-flowered, lanceolate, subterete; ***glumes*** shorter to as long as the spikelet, 1-veined, hyaline or sometimes membranous; ***lower glumes*** 0.25–0.5 as long as upper glumes; ***upper glumes*** 0.5–1 as long as the floret, apex acute to obtuse; ***lemmas*** 1–2.5 mm long, 1- or 3-veined, ovate, membranous, apex obtuse to acute; ***paleas*** 2-veined. ***Flowers*** perfect; ***lodicules*** 2; ***anthers*** 0.7–1.9 mm long; ***ovary*** glabrous. ***Caryopses*** 0.8–1.1 mm long, ellipsoid to globose, brownish with a free pericarp.

#### Species.

*Sporobolusacinifolius*, *S.albicans*, *S.tenellus*.

#### Distribution.

Southern Africa.

### ﻿ Sporobolus sect. Thellungia

Taxon classificationPlantaePoalesPoaceae

(Stapf) P.M.Peterson, Romasch. & Soreng, comb. et stat. nov.

8467329F-75BD-5297-B1E7-599B0E283DE0

urn:lsid:ipni.org:names:77367032-1

#### Type.

*Sporobolusadvenus* (Stapf) P.M. Peterson, Taxon 63(3): 1232. 2014 ≡ *Thellungiaadvena* Stapf ≡ *Eragrostisadvena* (Stapf) S.M. Phillips.

#### Description.

Caespitose ***perennials*** often with short to long rhizomes; innovations mostly intravaginal. ***Culms*** 60–300 cm tall, erect, sometimes geniculately ascending. ***Leaf sheaths*** open for most of their length, glabrous, sometimes coriaceous near base; ***ligules*** 0.1–0.8 mm long, a line of hairs; ***blades*** 10–30 cm long, (0.5–) 1–6 mm wide, flat to involute, sometimes convolute and filiform, smooth, scabrous near the margins, glabrous. ***Inflorescence*** a panicle 5–50 cm long, spiciform and contracted (*S.advenus*, *S.megalospermus*, sometimes *S.ramigerus*) or open (*S.collinus*), diffuse, ovate. ***Spikelets*** 3–17 mm long, 2–15 (–27)-flowered, linear, lanceolate, ovate to oblong, occasionally cleistogamous, sometimes with a rachilla extension (*S.advenus*); ***glumes*** shorter than the spikelet, 1-veined; ***lower glumes*** 0.5–0.9 as long as the upper glume; ***upper glumes*** 1.25–4 mm long, apex acute to obtuse; ***lemmas*** 1.8–3 mm long, 3-veined, sometimes 1-veined, ovate or lanceolate, hyaline, membranous to cartilaginous, often olive-green to dark greenish purple, apex acute, sometimes obtuse to truncate; ***paleas*** 1/2 to as long as the lemma, 2-veined, with wide flaps usually wider than the body and usually splitting along the midline; ***Flowers*** perfect; ***lodicules*** 2, cuneate; ***stamens*** 3, ***anthers*** 0.3–2 mm long, usually greenish; ***ovary*** glabrous. ***Caryopses*** 0.9–1.2 mm long, 3- or 4-angled, strongly laterally compressed to ovoid, globose, sometimes stipitate (*S.ramigerus*) with a free pericarp.

#### Species.

*Sporobolusadvenus*, *S.megalospermus*, *S.ramigerus*, and one more below.

#### Distribution.

Australasia and Central Asia.

### ﻿ Sporobolus collinus

Taxon classificationPlantaePoalesPoaceae

(Trin.) P.M.Peterson, Romasch. & Soreng, comb. nov.

7299AA06-2B47-5FD5-8B88-F590BFC51515

urn:lsid:ipni.org:names:77367031-1

Eragrostis collina Trin., Mém. Acad. Imp. Sci. St.-Pétersbourg, Sér. 6, Sci. Math. 1(4): 413. 1830 ≡ Poacollina (Trin.) K. Koch, Linnaea 21(4): 405. 1848, nom. illeg. hom., non Poacollina Host. ≡ Poatatarica Fisch. ex Griseb., Bess. Cat. Krzem. Suppl. 2: 13. 1814, nom. nud. ≡ Eragrostistatarica (Fisch. ex Griseb.) Nevski, Trudy Bot. Inst. Akad. Nauk S.S.S.R., Ser. 1, Fl. Sist. Vyssh. Rast.: 226. 1937 ≡ Eragrostistatarica (Fisch. ex Griseb.) Henrard, Blumea 3(3): 425. 1940, isonym. Type: Persia, E deserto Rhymnico, Hbr. Gorenki, *F.E.L. Fischer s.n.* (lectotype, designated here: LE-TRIN-2319.06 [microfiche image!]).
= Airaarundinacea L., Sp. Pl. 1: 64. 1753 ≡ Festucaarundinacea (L.) Lilj., Utkast Sv. Fl. (ed. 2): 47. 1798, hom. Illeg, non Festucaarundinacea Schreb. ≡ Poaarundinacea (L.) Link, Hort. Berol. [Link] 1: 176. 1827 ≡ Eragrostisarundinacea (L.) Roshev., Fl. SSSR 2: 319. 1934 ≡ Boriskelleraarundinacea (L.) Terechov, Del. Sem. Hort. Reg. Bot. Kujbyshev: 13. 1938 ≡ Psilanthaarundinacea (L.) Tzvelev, Bot. Zhurn. (Moscow & Leningrad) 53: 311. 1968. Type: Turkey, Aras Valley, 1100–1200 m, 19 Jul 1966, *P.H. Davis 46876* (neotype, designated by S.A. Renvoize in Cafferty et al. 2000, Taxon 49(2): 244: K; isoneotype: E, US-2597835!).

#### Notes.

We are rejecting the earlier lectotype by Tzvelev (1976) in Zlaki SSSR 635. Nauka Publishers, Leningrad Section, Leningrad, designating *Snowitz 518* as the lectotype for *Eragrostiscollina* because it was not mentioned by Trinius in the original publication.

## ﻿Discussion

The *E.collina* + (*S.ramigerus* (*S.advenus* + *S.megalospermus*)) clade is a biogeographically defined lineage occurring in Australia (*S.advenus*, *S.megalospermus*, and *S.ramigerus* are endemic to southern Australia, including Queensland and New South Wales) and extending to central and southwest Asia (Lazarides 1997; Palmer et al. 2005). *Eragrostiscollina* is endemic to Iran, Iraq, Crimea, Transcaucasia, central Asia around the Caspian and Aral seas to Kazakhstan and Kyrgyzstan, Turkistan, Uzbekistan (Syr-Darya), China (Xinjiang), Russia (Volga River basin from Dagestan to Siberia), and eastern Syria and Turkey (Bor 1970; Tan 1985; Palmer et al. 2005; Chen and Peterson 2006). Morphologically, these three species share multi-flowered spikelets with 2–27 florets and 3-veined (occasionally 1-veined) lemmas, two characteristics that are uncommon among species attributed to *Sporobolus*. The plastid lineage (PLC1) of *Eragrostiscollina* may have been donated through a hybridization event involving an unknown, extinct ancient lineage characterized by ancestral states (e.g., multi-flowered spikelets) from dry areas of eastern Asia, whereas the ITS (ITSC) signal clearly aligns as sister to *S.advenus* + *S.megalospermus*, all with an Australasian biogeography (Fig. 2A). Conversely, *S.advenus* and *S.megalospermus* may have received their plastid lineage from a presumed species in the American clade (Fig. 2A). In Australia, *S.advenus* and *S.megalospermus* are found in disturbed alluvial flats and other disturbed sites, which could have facilitated a chloroplast capture event from an American donor. Based on their ITS and morphological similarities, we place the four species in S.sect.Thellungia and transfer *E.collina* to *Sporobolus*.

Resolution among species sharing ITS and plastid genes between S.sect.Crypsis and S.sect.Virginici is more challenging. Based on ITS, the *S.consimilis* + *S.robustus* pair (ITSA), both primarily confined to Africa, shows that the latter species received its plastid haplotype (PLA1, B1) from a species probably within S.sect.Virginici, while *S.consimilis* received its plastid haplotype (PLA2) from an ancient hybridization event with a probable member of S.sect.Crypsis. Sporobolushumilissubsp.minor, sister to *S.mitchellii* (Trin.) C.E. Hubb. in S.sect.Crypsis (ITSB), subsect. Helvoli P.M. Peterson, received its plastid haplotype from a member of S.sect.Virginici, as it is sister to *S.robustus* (PLA1, B1). Previously, Peterson et al. (2014a) included S.humilissubsp.minor and *S.robustus* in S.sect.Virginici due to shared morphological characteristics such as narrow, densely spikeleted panicles or densely spikeleted primary branches (*S.robustus*), upper glumes longer than the floret, and stoloniferous growth (Baaijens and Veldkamp 1991). However, *S.consimilis* is still retained as *incertae sedis*, since it never forms a clade within S.sect.Crypsis, although plastid markers place it as sister to S.sect.Crypsis (PLA2) (Fig. 2A). Morphologically, *S.consimilis* is a large tussock-forming perennial with short underground rhizomes, sometimes forming looping stolons. Its panicles have spike-like primary branches bearing closely appressed spikelets, and the upper glumes are longer than the floret (Clayton 1974).

The three species representing S.subsect.Subulati–*S.kentrophyllus* + (*S.subulatus* − *S.verdcourtii*)–are evidently reticulate in origin. They resolve within S.sect.Pyramidati based on plastid data, with their plastid donor (PLE) likely a species within S.subsect.Pyramidati P.M. Peterson, where *S.ioclados* (Nees ex Trin.) Nees was placed. However, based on ITS, the same clade (ITSE) is sister to S.subsect.Actinocladi (Fig. 2B). These three species share the following morphological traits: caespitose perennials, often stoloniferous; panicles with whorled primary branches bare on the lower one-quarter to one-half; lower glumes one-third to three-quarters the length of the spikelets; upper glumes two-thirds to equal the length of the spikelet; and ellipsoid caryopses 0.8–2 mm long (Peterson et al. 2014a). They occur from Africa to India, with *S.kentrophyllus* and *S.verdcourtii* sometimes placed within *S.ioclados* (Plants of the World Online 2025).

The Australian species *S.scabridus* most likely received its plastid haplotype (PLF) from a member of S.subsect.Actinocladi but possesses an ITS marker similar to other members of S.subsect.Pyramidati (ITSF) (Fig. 2B). Morphologically, *S.scabridus* resembles *S.actinocladus* (F. Muell.) F. Muell., *S.caroli* Mez, and *S.coromandelianus* (Retz.) Kunth; all four species have quadrangular caryopses that are smooth on the ventral surface, with an embryo about half the length of the grain (Clayton 1974; Simon and Jacobs 1999). Species with African origins are found in both subsects *Actinocladi* and *Pyramidati*, increasing the likelihood of hybridization–particularly with the widespread *S.coromandelianus*.

Based on either plastid or ITS markers, *Sporobolustourneuxii* does not align within any existing section of *Sporobolus* (Fig. 2B). Broader sampling may reveal an affiliation with existing sections or subsections; otherwise, a new section may be warranted. *Sporobolustourneuxii* resembles *S.respolianus* Chiov. morphologically, both having short and stout panicle branches with spikelets crowded near the tips. However, the latter species has creeping stolons (Cope 1995; Phillips 1995). Both species occur along the Indian Ocean coastlines, overlapping in the Arabian Peninsula and the Horn of Africa.

Within the North American clade, *Sporobolusozarkanus* most likely received its plastid haplotype (PLG) from *S.splendens*, its strongly supported sister in S.sect.Airoides (Fig. 2B). Based on ITS, *S.ozarkanus* (ITSG) is sister to *S.clandestinus* + *S.vaginiflorus*. *Sporobolusozarkanus*, originally described by Fernald (1933) and later placed as a variety of *S.vaginiflorus* by Shinners (1954), is morphologically distinct from *S.vaginiflorus* by having sparsely hairy sheath bases, glumes longer than the florets, and 3-veined lemmas (Peterson et al. 2003). *Sporobolusozarkanus*, *S.clandestinus*, and *S.vaginiflorus* are found in the central and eastern United States and eastern Canada, whereas *S.splendens* is native to central and southern Mexico (Espejo Serna et al. 2000; Peterson et al. 2003).

We chose to recognize *S.compactus* and *S.somalensis* in a new genus, *Hyalolemma*, within the Sporobolinae, since together they are separated by a long branch indicating extensive genetic divergence from *Psilolemma*. Morphologically, *Psilolemma* differs from *Hyalolemma* by having a spiciform inflorescence composed of racemes arranged along a central axis; spikelets 3–3.8 mm long, 4–14-flowered; glumes membranous; lemmas 3–3.8 mm long with three conspicuous dark green veins, membranous; and caryopses 1.1–1.2 mm long (Phillips 1974). Both genera are endemic to the Horn of Africa (Fish et al. 2015).

Although our sampling of *Sporobolus* species is incomplete, we can make general observations about the biogeographical history of these grasses based on their current distribution. Our phylogeny suggests that *Sporobolus* originated in Africa, since *Psilolemma*, *Hyalolemma*, and S.sect.Acinifolii (all basal lineages) are from that region. The nearest sister group to the Sporobolinae–*Urochondra* (Somalia, Sudan to NW India) + *Zoysia* (temperate and tropical Asia, Australasia)–are members of the Zoysieae (Cope 1995; Phillips 1995; Clayton et al. 2006). The Zoysieae clade has an estimated mean crown age of 19.27 (14–25.23) mya and stem age of 36.82 (34.5–39.68) mya (Gallaher et al. 2022). The ancestral area estimate for Zoysieae (stem) is Afrotropical (46%) and Neotropics + Afrotropics (11%) (Gallaher et al. 2022).

As a worldwide genus, *Sporobolus* represents a rather “tight” evolutionary entity, with core species (those arising via orderly evolutionary descent rather than reticulate evolution) comprising about 81% of all sampled species (55% of the genus). This is significantly higher than in other widespread genera such as *Calamagrostis*–64% (57% sampled; Peterson et al. 2021) or *Agrostis*–55% (70% sampled; Peterson et al. 2025). In this respect, *Sporobolus* is closer to more localized genera like *Muhlenbergia*, with 96% core species (82% sampled; Peterson et al. 2010b, 2018), or *Bouteloua*, with 77% core species (98% sampled; Peterson et al. 2015b).

The phylogeography of S.sect.Thellungia resembles that of Australian *Agrostis* (Phase VI of phylogeographic distribution of *Agrostis*; Peterson et al. 2025). It includes the interaction (likely in dry areas of East Asia) of one of the most ancient, multiflowered Asian lineages–“proto-*collinus*”–and two derived, uniflowered, closely related but independent North American lineages–“proto-*megalospermus*” and “proto-*advenus*”–followed by the distribution of hybrids (all multiflowered) in Central Asia and Australia and their subsequent speciation. The origin of S.sect.Crypsis is likely African, although its paternal lineage remains unknown.

The involvement of species from S.sect.Triachyrum in the formation of sects. *Crypsis* and *Thellungia* is also possible. However, since no plastid lineages from *Triachyrum* species were found within these sections, we believe that *Crypsis* and *Thellungia* indeed represent floating ITS groups, and their sister placement to members of S.sect.Triachyrum is likely incidental, resulting from confounding ITS sequences.

## Supplementary Material

XML Treatment for Hyalolemma

XML Treatment for Hyalolemma compactum

XML Treatment for Hyalolemma somalensis

XML Treatment for Sporobolus sect. Acinifolii P.M.Peterson, Romasch. & Soreng, sect. nov.

XML Treatment for Sporobolus sect. Thellungia

XML Treatment for Sporobolus collinus

## ﻿Additional information

### Use of AI

No use of AI was reported.

### Author ORCIDs

Paul M. Peterson https://orcid.org/0000-0001-9405-5528

Konstantin Romaschenko https://orcid.org/0000-0002-7248-4193

Robert J. Soreng https://orcid.org/0000-0002-8358-4915

Yolanda Herrera Arrieta https://orcid.org/0000-0003-3814-6518

### Data availability

All of the data that support the findings of this study are available in the main text.

## ﻿Appendix 1

**Table A1.** List of specimens sampled. Taxon, voucher (collector, number, and where the specimen is housed), country of origin, specific data set, and GenBank accession for DNA sequences of *rps16–trnK* spacer, *rps16* intron, *rpl32–trnL* spacer, *ndhA* intron, and ITS regions; bold indicates new accession; a dash (–) indicates missing data.

N	Taxon	Voucher	Country	Data set	rps16–trnK	rps16	rpl32–trnL	ndhA	ITS
1	*Eragrostiscollina* Trin.	*Davis 46876* (USZ)	Turkey, A9 Kari	2A	**PV733880**	**PV733884**	**PV733875**	–	**PV714800**
2	*Eragrostiscollina* Trin.	*Field 303 & Lazar* (US)	Iraq	2A	**PV733881**	**PV733885**	**PV733876**	–	**PV714801**
3	*Eragrostiscollina* Trin.	*Nikitin 368* (US)	Kazakhstan, Aqtöbe	2A	**PV733882**	**PV733886**	**PV733877**	–	**PV714802**
4	*Eragrostiscollina* Trin.	*Rechinger 1048* (US)	Iran	2A	**PV733883**	**PV733887**	**PV733878**	–	**PV714803**
5	*Psilolemmajaegeri* (Pilg.) S.M. Phillips	*Peterson 24247, Soreng, Romaschenko & Mbago* (US)	Tanzania, Manyara	1, 2A, 2B	KM011122	KM010919	KM010695	KM010535	KM010326
6	*Psilolemmajaegeri* (Pilg.) S.M. Phillips	*Peterson 24249, Soreng, Romaschenko & Mbago* (US)	Tanzania, Manyara	1, 2A, 2B	KM011123	KM010920	KM010696	KM010536	KM010327
7	*Sporobolusacinifolius* Stapf	*Smook 3530* (US)	South Africa, Northern Cape	1, 2A, 2B	KM011153	KM010947	KM010727	KM010560	KM010354
8	*Sporobolusactinocladus* (F. Muell.) F. Muell.	*Saarela 1670, Peterson, Soreng & Judziewicz* (US)	Australia, Northern Territory	1, 2A, 2B	KM011155	KM010950	KM010730	KM010563	KM010357
9	*Sporobolusaculeatus* (L.) P.M. Peterson	*Soreng 5469, Peterson & Sun Hang* (US)	China, Gansu	2A	GU360599	GU360402	GU359841	GU359362	GU359238
10	*Sporobolusaculeatus* (L.) P.M. Peterson	*Soreng 7940, Johnson, Johnson, Dzyubenko, Dzyubenko & Schilnikov* (US)	Russia, Stavropol	2A	JQ345233	JQ345275	JQ345316	JQ345205	JQ345163
11	*Sporobolusacuminatus* (Trin.) Hack.	*Guala 1372 & Filgueiras* (US)	Brazil, Goias	1, 2A, 2B	KM011157	KM010952	KM010732	KM010565	KM010359
12	*Sporobolusadvenus* (Stapf) P.M. Peterson	*Belson s.n.* (US)	Australia, Queensland	2A	KM011303	–	KM010904	–	KM010522
13	*Sporobolusadvenus* (Stapf) P.M. Peterson	*Lazarides 4185* (US)	Australia, Queensland	2A	KM011304	–	KM010905	–	KM010523
14	*Sporobolusaeneus* (Trin.) Kunth	*Irwin 25327, Onishi, da Fonseca, Souza, Reis dos Santos & Ramos* (US)	Brazil, Goias	1, 2A, 2B	KM011159	KM010954	KM010734	–	KM010361
15	*Sporobolusafricanus* (Poir.) Robyns & Tournay	*Peterson 24024, Soreng, Romaschenko & Abeid* (US)	Tanzania, Njomba Region	1, 2A, 2B	KM011160	KM010955	KM010735	KM010567	KM010362
16	Sporobolusairoidessubsp.airoides (Torr.) Torr.	*Peterson 24587 & Romaschenko* (US)	Mexico, San Luis Potosí	1, 2A, 2B	KM011163	KM010958	KM010738	KM010570	KM010365
17	*Sporobolusalbicans* Nees	*Smook 2459 & Russell* (US)	South Africa, Orange Free State	1, 2A, 2B	–	–	KM010742	–	KM010369
18	*Sporobolusalopecuroides* (Piller & Mitterp.) P.M. Peterson	*Soreng 7941, Johnson, Johnson, Dzubenko & Schilnikov* (US)	Russia, Stavropol	2A	KM011116	KM010916	KM010688	KM010532	KM010320
19	*Sporobolusalterniflorus* (Loisel.) P.M. Peterson & Saarela	*Lakela 26537* (US)	USA, Florida	1, 2A, 2B	KM011127	KM010923	KM010700	KM010539	KM010330
20	*Sporobolusanglicus* (C.E. Hubb.) P.M. Peterson & Saarela	*Williams 2004-1* (UBC)	Canada, British Columbia	1, 2A, 2B	KM011130	KM010926	KM010703	KM010542	KM010333
21	*Sporobolusarcuatus* (K.E. Rogers) P.M. Peterson	*Rogers 42409, Sharp & Delgadillo* (US)	USA, Tennessee.	1, 2A, 2B	KM011110	KM010912	KM010683	KM010528	KM010315
22	*Sporobolusarenicola* P.M. Peterson	*Gates 17021* (US)	USA, Kansas.	1, 2A, 2B	KM011113	KM010913	KM010685	KM010529	KM010317
23	*Sporobolusatrovirens* (Kunth) Kunth	*Peterson 24729, Romaschenko & Zamudio Ruiz* (US)	Mexico, Queretaro	1, 2A, 2B	KM011170	KM010965	KM010747	KM010576	KM010374
24	*Sporobolusaustralasicus* Domin	*Peterson 14404, Soreng & Rosenberg* (US)	Australia, Western Australia	1, 2A, 2B	KM011172	KM010967	KM010749	KM010578	KM010376
25	*Sporobolusberteroanus* (Trin.) Hitchc. & Chase	*Peterson 8753, Annable & Poston* (US)	Ecuador, Cotopaxi	1, 2A, 2B	KM011174	–	KM010751	–	KM010378
26	*Sporobolusblakei* De Nardi ex B.K. Simon	*Latz 10662* (MEL)	Australia, Northern Territory	1, 2A, 2B	KM011175	KM010969	KM010752	KM010580	KM010379
27	*Sporobolusbogotensis* Swallen & García-Barr.	*Peterson 14970 & Refulio Rodriguez* (US)	Peru, Cajamarca	1, 2A, 2B	KM011176	KM010970	KM010753	–	KM010380
28	*Sporobolusbrevipilis* (Torr.) P.M. Peterson	*Strong 848, Kelloff, Schuyler, Wurdack & Churchill* (US)	USA, New Jersey	1, 2A, 2B	KM011111	–	KM010684	–	KM010316
29	*Sporobolusbrockmanii* Stapf	*Gillett 4016* (US)	Somalia, Hargesia	1, 2A, 2B	KM011177	KM010971	KM010754	KM010581	KM010381
30	*Sporobolusbuckleyi* Vasey	*Lira 546, Martinez, Alvarez, Ramirez, Medrod & Gamboa* (CIIDIR)	Mexico, Campeche	1, 2A, 2B	KM011178	KM010972	KM010755	KM010582	KM010382
31	*Sporoboluscaroli* Mez	*Speck 1915* (US)	Australia, Queensland	1, 2A, 2B	KM011183	KM010977	KM010760	KM010585	KM010387
32	*Sporoboluscentrifugus* (Trin.) Nees	*Hoener 2133* (US)	South Africa, Lesotho	1, 2A, 2B	KM011184	KM010978	KM010761	KM010586	KM010388
33	*Sporobolusclandestinus* (Biehler) Hitchc.	*Freeman 6687* (US)	USA, Kansas	1, 2A, 2B	KM011185	KM010979	KM010762	KM010587	KM010389
34	*Sporoboluscoahuilensis* Valdés-Reyna	*Peterson 10000, Annable & Valdes-Reyna* (US)	Mexico, Coahuila	1, 2A, 2B	KM011190	KM010984	KM010767	KM010591	KM010393
35	*Sporoboluscompactus* Clayton	*Boaler B17* (US)	Somalia	1, 2A, 2B	–	KM011075	KM010873	KM010654	KM010491
36	*Sporoboluscompositus* (Poir.) Merr.	*Brodovich 1305* (US)	USA, Michigan	1, 2A, 2B	KM011168	–	KM010745	KM010574	KM010372
37	*Sporobolusconfinis* (Steud.) Chiov.	*Peterson 24303, Soreng, Romaschenko & Mbago* (US)	Tanzania, Arusha	1, 2A, 2B	KM011191	KM010985	KM010768	KM010592	KM010394
38	*Sporobolusconsimilis* Fresen.	*Peterson 24252, Soreng, Romaschenko & Mbago* (US)	Tanzania, Manyara	2A	KM011193	KM010987	KM010771	KM010595	KM010396
39	*Sporoboluscontractus* Hitchc.	*Perez 196* (CIIDIR)	Mexico, Nuevo Leon	1, 2A, 2B	KM011194	KM010988	KM010772	–	KM010397
40	*Sporoboluscordofanus* (Hochst. ex Steud.) Coss.	*Laegaard 15973* (US)	Zimbabwe	1, 2A, 2B	KM011195	KM010989	KM010774	KM010596	KM010399
41	*Sporoboluscoromandelianus* (Retz.) Kunth	*Peterson 24269, Soreng, Romaschenko & Mbago* (US)	Tanzania, Shinyanga	1, 2A, 2B	KM011198	KM010992	KM010777	KM010598	KM010402
42	*Sporoboluscreber* De Nardi	*Brown 498* (MEL)	Australia, New South Wales	1, 2A, 2B	KM011200	KM010994	KM010779	KM010600	KM010404
43	*Sporoboluscryptandrus* (Torr.) A. Gray	*Peterson 22003 & Saarela* (US)	Mexico, Chihuahua	1, 2A, 2B	GU360631	GU360354	GU359914	GU359524	GU359208
44	*Sporoboluscynosuroides* (L.) P.M. Peterson & Saarela	*Hill 15630* (US)	USA, Maryland	1, 2A, 2B	KM011136	KM010931	KM010709	KM010546	KM010339
45	*Sporobolusdiandrus* (Retz.) P. Beauv.	*Peterson 14389, Soreng & Rosenberg* (US)	Australia, Western Australia	1, 2A, 2B	KM011203	KM010997	KM010782	KM010603	KM010407
46	*Sporobolusdinklagei* Mez	*Hale 11* (US)	Liberia, Cavalla Plantation	1, 2A, 2B	–	KM010998	KM010784	KM010604	KM010409
47	*Sporobolusdomingensis* (Trin.) Kunth	*Swallen 10669* (US)	USA, Florida	1, 2A, 2B	KM011205	KM010999	KM010785	KM010605	KM010410
48	*Sporoboluseylesii* Stent & J.M. Rattray	*Wiehe 717* (US)	Malawi, Nyasaland	1, 2A, 2B	–	KM011000	KM010787	–	KM010411
49	*Sporobolusfarinosus* Hosok.	*Wood 3275 & Perlman* (US)	Guam, Mariana Isl.	1, 2A, 2B	KM011206	KM011001	KM010788	KM010606	KM010412
50	*Sporobolusfertilis* (Steud.) Clayton	*Gould 13535* (US)	Sri Lanka	1, 2A, 2B	KM011207	KM011002	KM010789	KM010607	KM010413
51	*Sporobolusfestivus* Hochst. ex A. Rich.	*Peterson 23853, Soreng, Romaschenko & Abeid* (US)	Tanzania, Lindi	1, 2A, 2B	KM011209	KM011004	KM010791	KM010608	KM010415
52	*Sporobolusfimbriatus* (Trin.) Nees	*Peterson 24241, Soreng, Romaschenko & Mbago* (US)	Tanzania, Tanga	1, 2A, 2B	KM011211	KM011006	KM010794	KM010610	KM010417
53	*Sporobolusflexuosus* (Thurb. ex Vasey) Rydb.	*Valdes-Reyna 2014 & Peterson* (CIIDIR)	Mexico, Coahuila	1, 2A, 2B	KM011214	KM011009	KM010796	KM010612	KM010420
54	*Sporobolusfoliosus* (Trin.) P.M. Peterson & Saarela	*Reeder 6652 & Reeder* (US)	Mexico, Baja California Sur	1, 2A, 2B	KM011138	KM010933	KM010711	KM010548	KM010341
55	*Sporobolusgiganteus* Nash	*Pase 2628* (US)	USA, New Mexico	1, 2A, 2B	KM011216	KM011011	KM010800	–	KM010422
56	*Sporobolusgreenwayi* Napper	*Greenway 12526* (US)	Tanzania, Monduli	1, 2A, 2B	–	KM011012	KM010801	KM010614	KM010423
57	*Sporobolushelvolus* (Trin.) T. Durand & Schinz	*Peterson 24217, Soreng, Romaschenko & Mbago* (US)	Tanzania, Tanga	2A	KM011218	KM011014	KM010803	KM010615	KM010425
58	*Sporobolusheterolepis* (A. Gray) A. Gray	*Davidse 1910A* (US)	USA, Iowa	1, 2A, 2B	KM011219	KM011015	KM010804	–	KM010426
59	*Sporobolushookerianus* P.M. Peterson & Saarela	*Lewis 78-1013* (CAN)	Canada, Alberta	1, 2A, 2B	KM011140	KM010935	KM010713	KM010550	KM010343
60	Sporobolushumilissubsp.minor Veldkamp	*Clayton 5879* (US)	Sri Lanka, Eastren Province	2A	KM011220	KM011016	KM010805	KM010616	KM010427
61	*Sporobolusindicus* (L.) R. Br.	*Peterson 22025 & Saarela* (US)	Mexico, Chihuahua	1, 2A, 2B	GU360630	GU360355	GU359913	GU359504	GU359209
62	*Sporobolusinfirmus* Mez	*Haines 332* (US)	Nigeria, Igboora	1, 2A, 2B	KM011222	KM011018	KM010807	KM010618	KM010429
63	*Sporobolusioclados* (Nees ex Trin.) Nees	*Smook 5920* (US)	South Africa, Orange Free State	1, 2A, 2B	KM011223	KM011019	KM010808	KM010619	KM010430
64	*Sporobolusjacquemontii* Kunth	*15902 Peterson & Valdes-Reyna* (US)	Mexico, Tamaulipas	1, 2A, 2B	KM011225	KM011021	KM010810	KM010621	KM010432
65	*Sporobolusjunceus* (P. Beauv.) Kunth	*Strong 2332* (US)	USA, Florida	1, 2A, 2B	KM011226	KM011022	KM010811	KM010622	KM010433
66	*Sporoboluskentrophyllus* (K. Schum. ex Engl.) Clayton	*Bogdan 3306* (US)	Kenya	2B	KM011228	KM011024	KM010813	KM010624	KM010435
67	*Sporoboluslasiophyllus* Pilg.	*Peterson 21879, Soreng & Sanchez Vega* (US)	Peru, Cajamarca	1, 2A, 2B	GU360629	GU360356	GU359912	GU359505	GU359210
68	*Sporoboluslaxus* B.K. Simon	*Simon 4166* (MEL)	Australia, Queensland	1, 2A, 2B	KM011232	KM011028	KM010817	KM010626	KM010438
69	*Sporoboluslinearifolius* Nicora	*Reitz 5292 & Klein* (US)	Brazil, Santa Catarina	1, 2A, 2B	–	KM011029	KM010818	–	KM010439
70	*Sporobolusludwigii* Hochst.	*Smook 2857* (US)	South Africa, Orange Free State	1, 2A, 2B	KM011233	KM011030	KM010819	KM010627	KM010440
71	*Sporobolusmarginatus* Hochst. ex A. Rich.	*Rattray 1654* (US)	Zimbabwe, Matopos Res. Station	1, 2A, 2B	–	KM011033	KM010823	KM010628	KM010442
72	*Sporobolusmaritimus* (C.E. Hubb.) P.M. Peterson & Saarela	*Fernández Casas 5537, Castroviejo, Muñoz Garmendia & Susanna* (US)	Morocco, Tetouan	1, 2A, 2B	KM011142	KM010937	KM010715	KM010552	KM010345
73	*Sporobolusmegalospermus* (F. Muell. ex Benth.) P.M. Peterson	*Blake 6966* (US)	Australia, Queensland	2A	KM011117	–	KM010689	–	KM010321
74	*Sporobolusmegalospermus* (F. Muell. ex Benth.) P.M. Peterson	*Lazarides 4215* (US)	Australia, Queensland	2A	KM011118	–	KM010690	–	KM010322
75	*Sporobolusmegalospermus* (F. Muell. ex Benth.) P.M. Peterson	*Lazarides 5647* (US)	Australia, Queensland	2A	KM011119	–	KM010691	–	KM010323
76	*Sporobolusmichauxianus* (Hitchc.) P.M. Peterson & Saarela	*Dirig 2812* (US)	USA, Indiana	1, 2A, 2B	KM011150	KM010944	KM010723	KM010557	KM010351
77	*Sporobolusmicroprotus* Stapf	*Laegaard 17894 & Traore* (US)	Senegal, Thies	2B	KM011235	KM011035	KM010824	KM010629	KM010443
78	*Sporobolusmitchellii* (Trin.) C.E. Hubb. ex S.T. Blake	*Forster 22301* (MEL)	Australia, Queensland	2A	KM011236	–	KM010826	–	KM010444
79	*Sporobolusmolleri* Hack.	*Peterson 23978, Soreng, Romaschenko & Abeid* (US)	Tanzania, Ruvuma	1, 2A, 2B	KM011252	KM011052	KM010848	KM010641	KM010463
80	*Sporobolusmontanus* (Hook. f.) Engl.	*Dusen 420* (US)	Cameroon	1, 2A, 2B	–	KM011036	KM010829	KM010630	KM010447
81	*Sporobolusmyrianthus* Benth.	*Gereau 3491, Lovett & Kayombo* (DSM)	Tanzania, Mbeya	1, 2A, 2B	KM011239	–	KM010830	–	KM010448
82	*Sporobolusnatalensis* (Steud.) T. Durand & Schinz	*Eddie 1141* (MEL)	Australia, Queensland	1, 2A, 2B	KM011240	KM011037	KM010831	KM010631	KM010449
83	*Sporobolusnealleyi* Vasey	*Peterson 17839, Valdes-Reyna & Hinton* (US)	Mexico, Nuevo León	1, 2A, 2B	KM011242	KM011039	KM010833	KM010632	KM010450
84	*Sporobolusnervosus* Hochst.	*Wood 2021* (US)	Yemen	1, 2A, 2B	KM011245	KM011042	KM010836	KM010633	KM010453
85	*Sporobolusniliacus* (Fig. & De Not.) P.M. Peterson	*Moran 29081* (US)	Mexico, Baja California	2A	–	–	**PV733879**	–	**PV714804**
86	*Sporobolusnitens* Stent	*Laegaard 15893* (US)	Zimbabwe, Bulowayo	1, 2A, 2B	KM011246	KM011043	KM010837	KM010634	KM010454
87	*Sporobolusozarkanus* Fernald	*Riggins 481* (US)	USA, Texas	2B	KM011247	KM011045	KM010840	KM010635	KM010456
88	*Sporoboluspalmeri* Scribn.	*Peterson 24862 & Romaschenko* (US)	Mexico, San Luis Potosí	1, 2A, 2B	KM011248	KM011046	KM010841	KM010636	KM010457
89	*Sporoboluspanicoides* A. Rich.	*Smook 9865* (US)	South Africa, Namibia	1, 2A, 2B	–	KM011050	KM010846	KM010640	KM010461
90	*Sporoboluspectinellus* Mez	*Fay 7131* (US)	Central African Republic, Bamingui–Bangoran	1, 2A, 2B	–	KM011051	KM010847	–	KM010462
91	*Sporoboluspellucidus* Hochst.	*Vesey-Fitzgerald 5226* (US)	Tanzania, Arusha	1, 2A, 2B	–	KM011053	KM010849	–	KM010464
92	*Sporobolusphleoides* Hack.	*Venturi 2039* (US)	Argentina, Tucuman	1, 2A, 2B	KM011253	KM011054	KM010850	–	KM010465
93	*Sporobolusphyllotrichus* Hochst.	*Greenway 11844 & Kanuri* (US)	Tanzania, Arusha	1, 2A, 2B	–	KM011055	KM010851	–	KM010466
94	*Sporoboluspiliferus* (Trin.) Kunth	*Peterson 24012, Soreng, Romaschenko & Abeid* (US)	Tanzania, Njombe	1, 2A, 2B	KM011254	KM011056	KM010852	KM010642	KM010467
95	*Sporoboluspinetorum* Weakley & P.M. Peterson	*Peterson 14233, Weakley & LeBlond* (US)	USA, North Carolina	1, 2A, 2B	KM011255	GU360358	GU359911	GU359506	GU359211
96	*Sporoboluspseudairoides* Parodi	*Wasum 2670* (US)	Brazil, Parana	1, 2A, 2B	KM011256	KM011057	KM010853	–	KM010468
97	*Sporoboluspumilus* (L.) P.M. Peterson & Saarela	*Shchepanek 6426 & Dugal* (CAN)	Canada, Nova Scotia	1, 2A, 2B	KM011148	KM010942	KM010721	KM010555	KM010349
98	*Sporoboluspungens* (Schreb.) Kunth	*Zohary 489 & Amdursky* (US)	Israel	1, 2A, 2B	KM011257	KM011058	KM010854	KM010643	KM010470
99	*Sporoboluspurpurascens* (Sw.) Ham.	*Swallen 10179* (US)	USA, Texas	1, 2A, 2B	KM011259	KM011060	KM010856	KM010645	KM010472
100	*Sporoboluspyramidalis* P. Beauv.	*Peterson 24150, Soreng, Romaschenko & Abeid* (US)	Tanzania, Iringa	1, 2A, 2B	KM011261	KM011062	KM010858	KM010646	KM010474
101	*Sporoboluspyramidatus* (Lam.) Hitchc.	*Peterson 18994, González-Elizondo, Carter, Rosen, Guaglianone & Torres Soto* (US)	Mexico, Durango	1, 2A, 2B	KM011265	KM011065	KM010861	KM010648	KM010478
102	*Sporobolusramigerus* (F. Muell.) P.M. Peterson, Romasch. & R.L. Barrett	*Peterson 14338, Soreng & Rosenberg* (US)		2A	MK872671	–	MK872424	–	MK863099
103	*Sporobolusramigerus* (F. Muell.) P.M. Peterson, Romasch. & R.L. Barrett	*Peterson 14357, Soreng & Rosenberg* (US)		2A	MK872672	–	MK872425	–	MK863100
104	*Sporobolusrigens* (Trin.) Desv.	*Peterson 19224, Soreng, Salariado & Panizza* (US)	Argentina, Mendoza	1, 2A, 2B	GU360627	GU360360	GU359909	GU359517	GU359213
105	*Sporobolusrigidus* (Buckley) P.M. Peterson	*Hatch 5738 & Bearden* (US)	USA, Colorado	1, 2A, 2B	GU360548	GU360441	GU359880	GU359357	GU359300
106	*Sporobolusrobustus* Kunth	*Laegaard 17398, Goudiaby, Madsen, Sambou & Traore* (US)	Senegal, Kaolack	2A	KM011268	KM011068	KM010864	KM010650	KM010481
107	*Sporobolusruspolianus* Chiov.	*McKinnon S91* (US)	Somalia, Erigavo	2A	KM011215	KM011010	KM010799	KM010613	KM010421
108	*Sporobolussanguineus* Rendle	*Gereau 6014, Mbago, Kayonbo & Lyanga* (DSM)	Tanzania, Kigoma	1, 2A, 2B	KM011272	–	KM010868	–	KM010485
109	*Sporobolusscabridus* S.T. Blake	*Forster 20462* (MEL)	Australia, Queensland	1, 2A, 2B	KM011273	KM011071	KM010869	KM010652	KM010486
110	*Sporobolusschoenoides* (L.) P.M. Peterson	*Peterson 19814, Saarela & Sears* (US)	USA, California	2A	GU360598	GU360455	GU359840	GU359361	GU359239
111	*Sporobolussessilis* B.K. Simon	*Senaratne E60951 & Armstrong* (US)	Australia, Queensland	1, 2A, 2B	KM011274	KM011073	KM010871	KM010653	KM010488
112	*Sporobolussilveanus* Swallen	*Waller 3128 & Bauml* (US)	USA, Texas	1, 2A, 2B	KM011275	KM011074	KM010872	–	KM010489
113	*Sporobolussomalensis* Chiov.	*Hemming 2022* (US)	Somalia	1, 2A, 2B	–	KM011076	KM010874	KM010655	KM010492
114	*Sporobolusspartinus* (Trin.) P.M. Peterson & Saarela	*Reeder 4568 & Reeder* (US)	Mexico, Coahuila	1, 2A, 2B	KM011151	KM010945	KM010724	KM010558	KM010352
115	*Sporobolusspicatus* (Vahl) Kunth	*Peterson 24055, Soreng, Romaschenko & Abeid* (US)	Tanzania, Mbeya	2B	KM011278	KM011079	KM010877	KM010658	KM010495
116	*Sporobolusspiciformis* Swallen	*Garcia 2638* (CIIDIR)	Mexico, Durango	1, 2A, 2B	KM011280	KM011081	KM010879	KM010660	KM010497
117	*Sporobolussplendens* Swallen	*King 1687 & Feddema* (US)	Mexico, Oxaca	1, 2A, 2B	KM011282	KM011083	KM010881	KM010662	KM010499
118	*Sporobolusstapfianus* Gand.	*Laegaard 15939* (US)	Zimbabwe	1, 2A, 2B	KM011283	KM011084	KM010882	–	KM010500
119	*Sporobolusstolzii* Mez	*Peterson 23946, Soreng, Romaschenko & Abeid* (US)	Tanzania, Ruvuma	1, 2A, 2B	KM011284	KM011085	KM010883	KM010663	KM010501
120	*Sporobolussubglobosus* Stapf ex C.E. Hubb.	*Gambaga 581* (US)	Ghana, Gambaga	1, 2A, 2B	–	KM011088	KM010885	–	KM010504
121	*Sporobolussubulatus* Hack.	*Peterson 24317, Soreng, Romaschenko & Mbago* (US)	Tanzania, Arusha	2B	KM011287	KM011089	KM010886	KM010666	KM010505
122	*Sporobolustenellus* (Spreng.) Kunth	*Smook 2874* (US)	South Africa, Orange Free State	1, 2A, 2B	–	KM011090	KM010887	KM010667	KM010507
123	*Sporobolustenuissimus* (Mart. ex Schrank) Kuntze	*Peterson 9523 & Judziewicz* (US)	Ecuador, Pichincha	1, 2A, 2B	KM011289	KM011091	KM010889	KM010669	KM010508
124	*Sporobolusteretifolius* R.M. Harper	*Peterson 14232, Weakley & LeBlond* (US)	USA, North Carolina	1, 2A, 2B	GU360626	GU360376	GU359908	GU359509	GU359199
125	*Sporobolustexanus* Vasey	*Churchill 2645 & Kaul* (US)	USA, Nebraska	1, 2A, 2B	KM011291	KM011093	KM010891	KM010671	KM010510
126	Sporobolus×townsendii (H. Groves & J. Groves) P.M. Peterson & Saarela	*Saarela 791 & Percy* (UBC)	Canada, British Columbia	1, 2A, 2B	KM011126	KM010922	KM010699	KM010538	KM010329
127	*Sporobolustourneuxii* Coss.	*Adam 19416* (US)	Mauritania	2B	KM011292	KM011094	KM010892	KM010672	KM010511
128	*Sporobolustrichodes* Hitchc.	*Rzedowski 39901* (CIIDIR)	Mexico, Michoacán	1, 2A, 2B	KM011293	KM011095	KM010893	KM010673	KM010512
129	*Sporobolusuniglumis* Stent & J.M. Rattray	*Robinson 48* (US)	Zambia	2B	–	KM011096	KM010894	–	KM010513
130	*Sporobolusvaginiflorus* (Torr. ex A. Gray) Alph. Wood	*Wherry s.n.* (US)	USA, Pennsylvania	1, 2A, 2B	KM011296	KM011099	KM010897	–	KM010515
131	*Sporobolusverdcourtii* Napper	*Vesey-Fitzgerald 5336* (US)	Kenya, Olagasale	2B	KM011297	KM011100	KM010898	KM010674	KM010516
132	*Sporobolusvirginicus* (L.) Kunth	*Peterson 23820, Soreng, Romaschenko & Abeid* (US)	Tanzania, Lindi	1, 2A, 2B	KM011299	KM011102	KM010900	–	KM010518
133	*Sporoboluswrightii* Munro ex Scribn.	*Peterson 19841 & Lara-Contreras* (US)	Mexico, Coahuila	1, 2A, 2B	GU360624	GU360348	GU359906	GU359511	GU359216
134	*Urochondrasetulosa* (Trin.) C.E. Hubb.	*Inckennon 181* (US)	Somalia	1, 2A, 2B	KM011307	KM011108	KM010908	KM010681	KM010526
135	*Zoysiajaponica* Steud.	*Kuragadake s.n.* (US)	Japan	1, 2A, 2B	GU360643	–	GU359923	GU359547	GU359196
